# Organophosphorus Flame Retardant TCPP Induces Cellular Senescence in Normal Human Skin Keratinocytes: Implication for Skin Aging

**DOI:** 10.3390/ijms232214306

**Published:** 2022-11-18

**Authors:** Jian-Xiang Liu, Dao-Lei Cui, Dan-Lei Yang, Jing-Ya Li, Zi-Yue Yang, Jin-Zhou Su, Cai-Xia Ren, You-Ya Niu, Ping Xiang

**Affiliations:** 1 Yunnan Province Innovative Research Team of Environmental Pollution, Food Safety and Human Health, Institute of Environmental Remediation and Human Health, School of Ecology and Environment, Southwest Forestry University, Kunming 650224, China; 2 School of Basic Medical Sciences, Hunan University of Medicine, Huaihua 418000, China

**Keywords:** TCPP, human skin keratinocytes (HaCaT), DNA damage, cell cycle arrest, cellular senescence, human skin aging

## Abstract

Tris (1-chloro-2-propyl) phosphate (TCPP) is one of the most frequently detected organophosphorus flames in the environment. Continuous daily exposure to TCPP may harm human skin. However, little is known about the adverse effects of TCPP on human skin. In this study, we first evaluated the detrimental effects and tried to uncover the underlying mechanisms of TCPP on human skin keratinocytes (HaCaT) after 24 h exposure. We found that TCPP caused a concentration-dependent decrease in HaCaT cell viability after exposure to 1.56–400 μg/mL for 24 h, with an IC_50_ of 275 μg/mL. TCPP also promoted the generation of intracellular reactive oxygen species (ROS) and triggered DNA damage, evidenced by an increase of phosphorylated histone H2A.X (γH2A.X) in the nucleus. Furthermore, the cell cycle was arrested at the G1 phase at 100 μg/mL by upregulation of the mRNA expression of *p53* and *p21* and downregulation of *cyclin D1* and *CDK4* expression. Additionally, both the senescence-associated-β-galactosidase activity and related proinflammatory cytokine IL-1β and IL-6 were elevated, indicating that TCPP exposure caused cellular senescence may be through the p53-dependent DNA damage signal pathway in HaCaT cells. Taken together, our data suggest that flame-retardant exposure may be a key precipitating factor for human skin aging.

## 1. Introduction

Tris (1-chloro-2-propyl) phosphate (TCPP) as an organophosphorus flame retardant (OPFR) has been widely used in various consumer products, building materials, and baby products [1,2]. Due to its material characteristics, the lack of covalent bonding makes it easy for TCPP to leach out of products over time to pollute the environmental media, like dust, and enter humans. TCPP has been frequently detected in indoor dust, with concentrations being 270–39,300 ng/g [3]. In addition, it has been observed in human serum, breast milk, and urine [4,5,6]. Therefore, the toxicity of TCPP has attracted the scientific community. Studies have been demonstrated that skin absorption may be an important route for human exposure to OPFR, including TCPP [7,8]. However, the adverse effects of TCPP on human skin are largely unknown.

The human skin is the largest body organ of the integumentary system, with a surface area of about 2 m^2^ and weighing about 5 kg in adult people [8]. However, unlike other organs, the skin is in direct contact with outside environmental factors, which may age as a result of environmental damage [9]. Skin aging is a complex process caused both by intrinsic and extrinsic factors [10]. The external skin aging process is mainly induced by a variety of factors, including ultraviolet rays, air pollution, oxidative stress, DNA damage, and chemical substances [11]. Environmental pollution is a recognized risk extrinsic factor for skin aging [12] and may involve any one of the thousands of chemicals, including TCPP. The outermost cell layer of human skin (epidermis) mainly consists of keratinocytes, which act as a barrier to protect the vascular dermis from exposure to contaminants. Keratinocytes are the major cell type impacted by TCPP penetrating the epidermis. The contaminants, including TCPP invading the epidermal cells (keratinocytes), could increase the generation of intracellular reactive oxygen species (ROS) and promote aging-related signal transduction, leading to cellular senescence. Cellular senescence is characterized by irreversible cell cycle arrest along with cell enlargement and vacuolation, up-regulation of involved genes, and secretion of proteins leading to the development of inflammation [10]. In addition, most of those actions were regulated by p53/p21 and/or p16-Rb pathway [13,14].

Mounting evidence demonstrated that flame retardant exposure may induce cellular senescence. Behnia et al. (2015) found that exposure to polybrominated diphenyl ethers (PBDEs) flame retardant could induce primary amnion cell senescence [15]. Furthermore, TCEP-induced cellular senescence via activation of the p21Waf1/Cip1-Rb pathway was also observed in the human L02 hepatocytes [16]. In addition, cellular senescence is also a source of inflammatory factors. The secretion of several bioactive molecules (e.g., *Il-6*) in senescent cells is known as senescence-associated secretory phenotype (SASP), and those bioactive molecules play an important role in the progress of senescence [17]. However, the adverse effects of TCPP exposure on human skin cells and its underlying mechanism are largely unknown.

In this study, to better understand its toxicity, normal human skin keratinocytes (HaCaT) were employed, and the changes of cell viability, morphology, reactive oxygen species (ROS), DNA damage factor Phosphorylated histone H2A.X (γH2A.X), cell cycle arrest, and senescence-associated-β-galactosidase activity (SA-β-gal) were determined after TCPP exposure. Additionally, the mRNA and protein expression of senescence markers was detected to uncover the underlying mechanism of TCPP-induced of skin aging.

## 2. Results and Discussion

### 2.1. TCPP Suppressed Cell Viability and Altered Cell Morphology 

**Figure 1:**
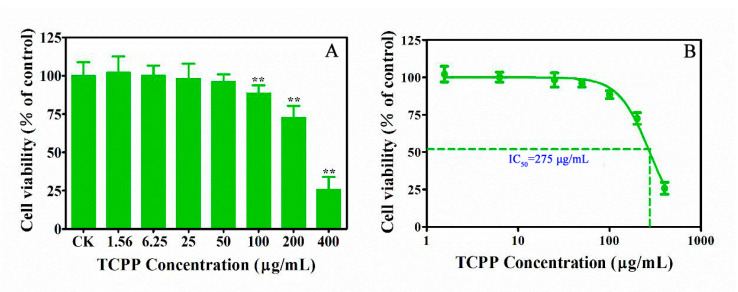
Cytotoxicity of TCPP on HaCaT cells after exposure to 1.56–400 µg/mL TCPP for 24 h (**A**) and logarithmic transformation of TCPP concentrations and cell viability data to determine IC_50_ (**B**). ** *p* < 0.01.

The skin is in direct contact with outside environmental factors, which usually leads to age at a cellular level as a result of environmental damage [12]. Changes in cellular viability and morphology have all been postulated to contribute to the aging process [18]. Cell viability is an important assay to screen cellular responses to contaminants, which is widely used to quantify cell proliferation and metabolic activities, and to assess cell senescence after exposure [10]. Based on the detected concentrations of TCPP in the environmental samples, including indoor dust with concentrations from 270 to 39,300 ng/g [3]. The effects of TCPP on the viability of HaCaT cells were determined using the CCK-8 assay according to our previous study [19]. After 24 h exposure, a toxic effect on HaCaT cell viability was observed at TCPP > 50 μg/mL (Figure 1A). At 100–200 μg/mL TCPP, cell viability was inhibited by 12–28% (Figure 1A), which was consistent with a previous study that TCPP at 164 μg/mL suppressed the cell viability up to 47.7% after 24 h exposure in human peripheral blood mononuclear cells [20], indicating that human skin epidermal cells were more susceptible to TCPP than human blood mononuclear cells [21]. Moreover, when TCPP exposure concentrations increased to 400 μg/mL, there was a sharp decrease in viable cells (74%), suggesting that exposure to TCPP at high concentrations could result in serious damage to human skin. The fitted curve shows that the IC_50_ concentrations of TCPP at 275 μg/mL (Figure 1B), which was lower than that of human peripheral blood mononuclear cells (328 μg/mL), consistent with lower toxicity of TCPP in human peripheral blood mononuclear cells [20]. 

**Figure 2:**
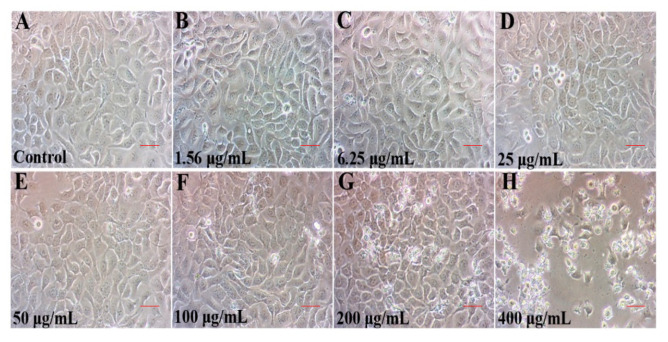
The morphology change (**A**–**H**) of HaCaT cells after exposure to 1.56–400 µg/mL TCPP for 24 h. Images were recorded under an inverted phase contrast microscopy at 200× magnification. Scale bar 50 μm.

In addition to cell viability, cellular morphology change is an important indicator of dysfunction of physiological function and cellular senescence [10]. The typical cobblestone and polygonal appearance of HaCaT cells were clear in the control (Figure 2A) and those exposed to TCPP at <100 μg/mL (Figure 2B–F). At ≥200 μg/mL, an irregular shape and an increased number of round and floating cells were observed (Figure 2G,H), implicating cellular senescence and death [22]. Taken together, our data implied that TCPP perturbed the monolayer morphology and inhibited cell proliferation of HaCaT cells, which is likely associated with cellular senescence (Figure 2F–H). However, further studies were warranted to confirm our hypothesis that TCPP exposure may trigger cellular senescence.

### 2.2. TCPP Increased Intracellular ROS, Induced DNA Damage and Cell Cycle Arrest in HaCaT Cells

**Figure 3:**
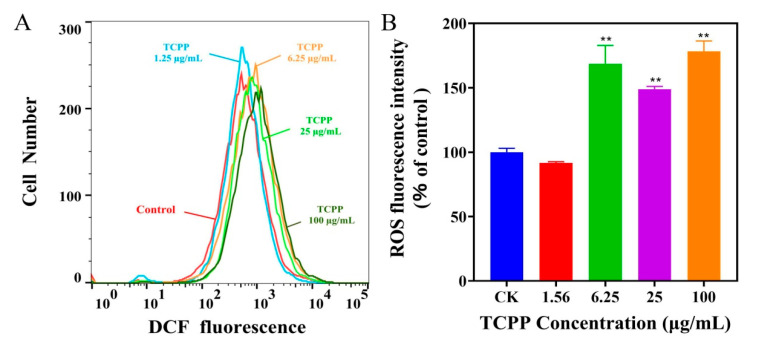
TCPP induced oxidative stress in HaCaT cells after 24 h exposure. ROS level was detected with DCFH-DA using a flow cytometer (**A**). The mean fluorescence intensity (MFI) was expressed as % of control (**B**). ** *p* < 0.01.

In addition to changes in cell viability and morphology, oxidative stress is also a crucial mechanism of flame retardants-induced cellular senescence [23], and oxidative stress is caused by excess ROS [24]. Saquib et al. (2019) found that flame retardants (6-OHBDE-47) can disrupt the mitochondrial potential, which promotes intracellular ROS generation in HepG2 cells [25]. Given that, we evaluated the levels of intracellular ROS after TCPP exposure at 1.56–100 μg/mL using the flow cytometer analysis with DCFH-DA fluorescent probe (Figure 3A). We found that the levels of intracellular ROS significant increase after exposure to TCPP at >1.56 μg/mL (Figure 3B). The ROS levels were elevated to 149–178% compared to the control after exposure to 6.25–100 μg/mL TCPP (Figure 3B), which is similar to Yang et al. (2022) showing that flame retardant (PBDEs) exposure increased ROS levels in J774A.1 cells [26].

DNA damage is another extensive feature of cellular senescence and aging [27]. It is well known that ROS-mediated DNA damage activates the p53 pathway, resulting in cellular senescence [28]. Accumulation of DNA damage, including γ-H2AX, is a major driver of premature senescence. To assess whether possible induction of DNA damage, we used a marker phosphorylated histone H2A.X (γH2A.X). H2A.X is a member of the histone H2A family; at its C-terminus, there is a highly conserved homologous sequence consisting of 22 residues, which can be phosphorylated after DNA damage occurs in somatic cells [29,30]. Subsequently, γH2A.X gathers in double-stranded breaks to form a large number of γ-H2A.X foci. Therefore, the foci assay of γH2A.X is a well-known indicator to evaluate the levels of DNA damage [31]. 

**Figure 4:**
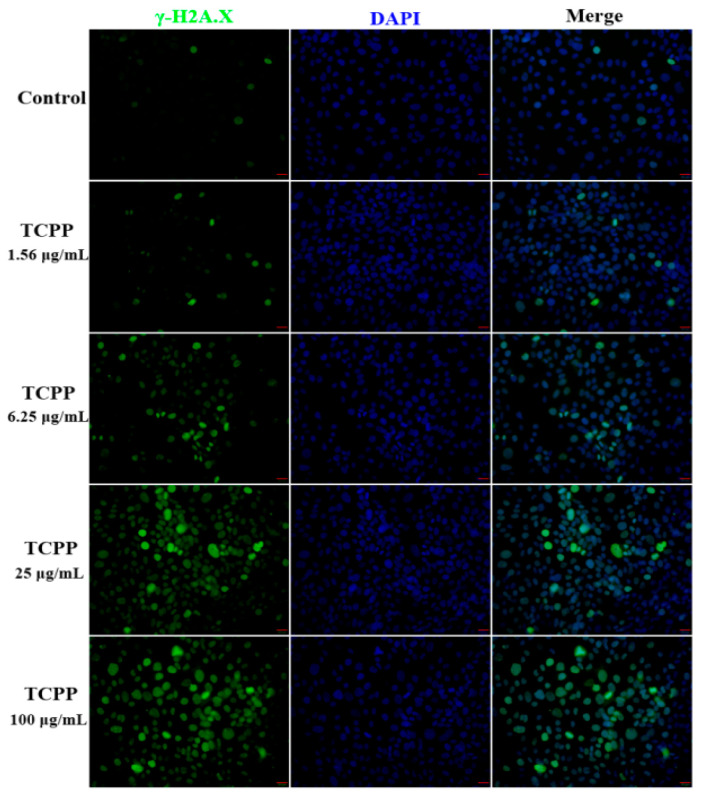
TCPP induced DNA damage in HaCaT cells after exposure to 1.56–100 μg/mL TCPP for 24 h. The green fluorescence intensity of γ-H2AX (green) was elevated by increasing TCPP concentration in HaCaT cells, suggesting aggravation of DNA damage. DAPI counterstains the nuclei (blue), 200× magnification. Scale bar 20 μm.

**Figure 5:**
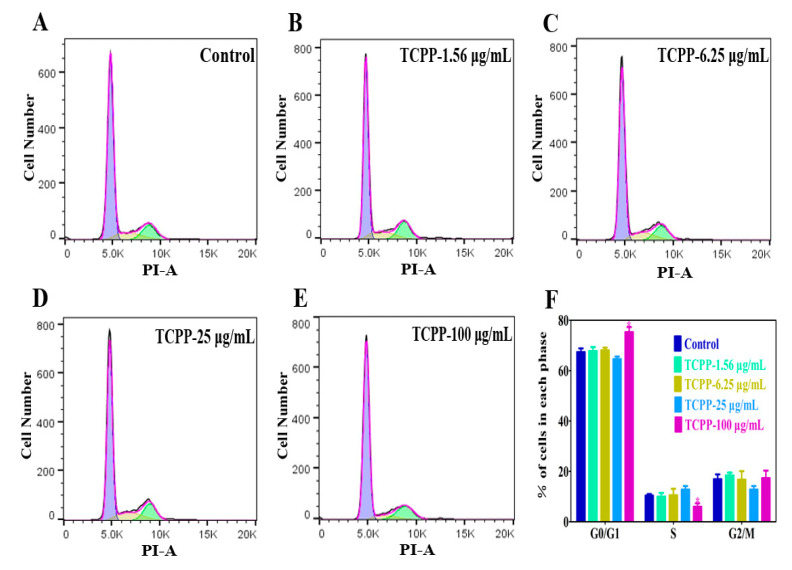
Cell cycle arrest was measured by flow cytometry after exposure to 1.56–100 μg/mL TCPP for 24 h (**A**–**E**) and the change of cell population induced by TCPP (**F**). TCPP caused a marked G0/G1 cell cycle arrest, evidenced by a higher number of cells residing in the G0/G1 phase and reducing S phase entry, especially at 100 μg/mL (* *p* < 0.05).

In this study, we used immunofluorescence to detect γ-H2A.X fluorescence after exposing TCPP at 1.56–100 μg/mL in HaCaT cells. We found that HaCaT cells exhibited elevated green fluorescence intensity in TCPP-treated cells in a concentration-dependent manner compared to the control (Figure 4). The percentage of fluoresced green reached 21 ± 5.8, 31 ± 3.2, 47 ± 6.3, and 73 ± 9.3% in HaCaT cells at 1.56, 6.25, 25, and 100 μg/mL (Figure 4), which was associated with the percentage of viability inhibition (Figure 1) and cell cycle arrest (Figure 5). The effect of TCPP on γH2AX expression showed a similar pattern to the decrease of cell viability in HaCaT cells, which is consistent with Yang et al. (2012), who showed that Di (2-Ethylhexyl) phthalate induced DNA damage in HepG2 cells [32].

In addition, cell cycle arrest is an important indicator of cell aging, so it has also been used for identifying cell aging [33]. The cell cycle consists of G1, S, and G2/M phases, and the activation of each phase depends on the correct progress and completion of the previous phase. Cui et al. (2020) showed that exposure to OPFR tris (1,3-dichloro-2-propyl) phosphate (TDCPP) at 16 μg/mL caused cell cycle arrest at the G1 phase in HaCaT cells [34]. However, it is unknown if this was the case with TCPP. The results show that the cell cycle of HaCaT cells did not change when TCPP at 1.56, 6.25, and 25 μg/mL (Figure 5A–D). At 100 μg/mL, HaCaT cells showed 76% were arrested in the G1 phase (Figure 5E,F), which was higher than the control at 67% (Figure 5A). Moreover, S-phase cells were reduced from 10 to 5% after exposing them to 100 μg/mL TCPP for 24 h (Figure 5E,F). The data suggest that TCPP induced cell cycle arrest at TCPP ≥ 100 μg/mL, which was consistent with the result of cell viability data (Figure 1A) and consistent with a previous report that TCPP treatment induced an obvious G1 phase cell cycle arrest in HepG2 cells in a concentration-dependent manner [35], indicating it may trigger skin aging.

### 2.3. TCPP Enhanced Cellular SA-β-Gal Activity in HaCaT Cells

**Figure 6:**
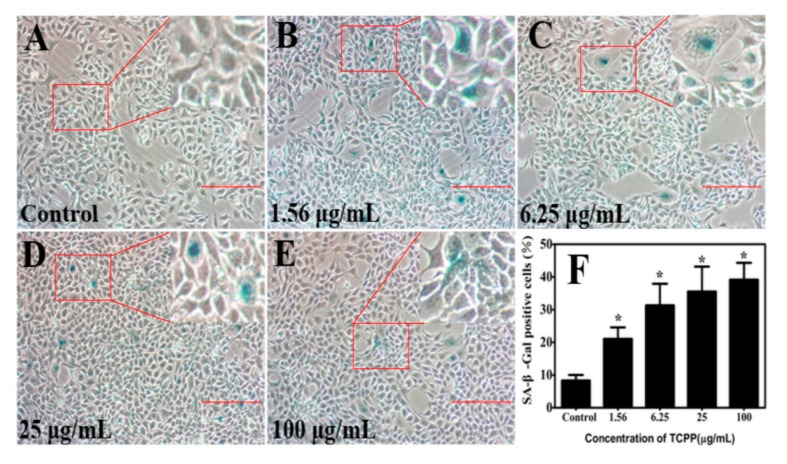
TCPP increased senescence markers (senescence-associated-β -galactosidase (SA-β-Gal) activity) in cultured HaCaT cells (**A**–**E**) after exposure to 1.56–100 µg/mL TCPP for 24 h (**A**–**E**), and the percentage of SA-β-Gal positive cells (blue color) was shown in (**F**). The senescence phenotype was seen as blue staining cells. 200× magnification. Scale bar 100 μm. * *p* < 0.05.

Cellular senescence is defined as an irreversible cell growth arrest that occurs in response to cellular stressors, including the decrease of cell viability, changes in cellular morphology, or DNA damage [36]. The most widely used assay for senescent and aging cell phenotypes is the histochemical detection of senescence-associated beta-galactosidase (SA-β-gal), which is known as SA-β-Gal activity. The SA-β-gal activity causes by an elevated transcription of GLB-1, the gene encoding the lysosomal beta-galactosidase (β-gal) [37]. The upregulation of the GLB-1 gene results from an increase in the number and activity of lysosomes, which is attributed to the accumulation of dysfunctional macromolecules in aging cells. The SA-β-gal activity was significantly related to the aging cells; however, it was not observed in terminally differentiated cells or quiescent cells [38]. Behnia et al. (2015) pointed out that flame retardant, polybrominated diphenyl ether-exposed cells exhibited morphologic changes with higher SA β-gal-stained cells than the control [15]. Zhang et al. (2017) demonstrated that flame retardant Tris (2-chloroethyl) phosphate (TCEP) exposure induced a senescence-like phenotype of hepatocytes, with an elevation of the percentage of SA-β-gal positive cells [16]. To validate whether TCPP at 1.56–100 μg/mL induced HaCaT cell senescence, the cellular SA-β-gal activity was detected after 24 h treatment. As shown in Figure 6A–F, TCPP triggered HaCaT cell senescence in a dose-dependent manner, as evidenced by strongly enhanced SA-β-gal activity, the percentages of SA-β-Gal positive cells were increased from 21–39% in TCPP-treated groups, which was in accord with a previous study that TCEP elevated the ratio of SA-β-Gal positive cells in hepatocytes from 20–37% with increased exposure levels (3.12–200 μg/mL) [16]. 

### 2.4. TCPP Altered Gene Expression of Senescence Markers

**Figure 7:**
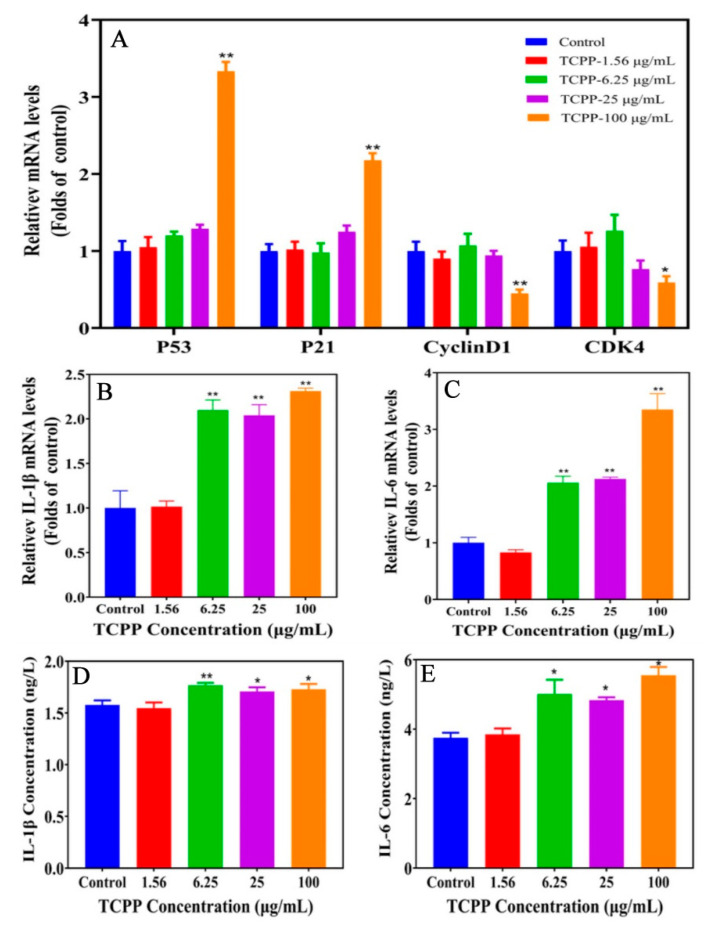
TCPP changed cell senescence and cycle (**A**) and SASP (**B**–**E**) regulatory gene expression of HaCaT cells after exposing them to 1.56–100 µg/mL TCPP for 24 h. * *p* < 0.05, ** *p* < 0.01.

To better understand the underlying molecular mechanisms of cell senescence, we determined the transcriptional expression of important mediators in the cell senescence process. The mounting evidence demonstrated that p53/p21 pathway has a key role in cellular senescence in various human cell lines, responding to a range of cellular damage signals [39,40,41]. It is known that cellular senescence was regulated by p53, a biomarker of DNA damage that responds to stressful stimuli through the p53 pathway. Studies show that p53 plays a key role in DNA damage response [42]. Its activation in response to DNA damage causes cell cycle arrest and cell growth inhibition, inducing cells to enter senescence [43]. In addition, p53 induces gene expression related to senescence, such as *p21*, preventing cell growth through cyclin-dependent kinase (CDK), leading to cell cycle arrest [13,44]. Therefore, up-regulation of *p53*/*p21* mRNA expression may be an important mechanism for contaminant-induced cellular senescence in human cells, including HaCaT cells. In this study, TCPP did not affect the mRNA expression of *p53* and *p21* at ≤25 μg/mL; however, when concentration increased to 100 μg/mL TCPP, the expression of *p53* and *p21* was increased to 3.4 and 2.2 folds, while *cyclinD1* and *CDK4* were decreased to 0.45 and 0.59 folds (Figure 7A). The data suggest that TCPP elicited G1 phase arrest in HaCaT cells via enhancing *p53* and *p21* and suppression of *cyclin D1* and *CDK4* expression.

In addition, senescence is also characterized by the secretion of cytokines known as the senescence-associated secretory phenotype (SASP) [17]. Cellular senescence is mediated by SASP involving proinflammatory cytokine secretion [45]. IL-1β and IL-6 are the main SASP proinflammatory cytokine for cellular senescence [16]. The exposure to 6.25–100 μg/mL TCPP enhanced IL-1β (2.1–2.3 folds) and IL-6 (2.1–3.3 folds) mRNA levels. Additionally, we further detected protein levels of IL-1β and IL-6 using commercial ELISA kits, and the result showed the levels of IL-1β (1.7–1.8 ng/L) and IL-6 (5–5.6 ng/L) were significantly increased compared with that of the control (IL-1β, 1.5 ng/L) and (IL-6, 3.7 ng/L) (Figure 7B–E). Taken together, our results indicated that TCPP exposure induces cellular senescence in HaCaT cells may be via the p53/p21 pathway.

## 3. Materials and Methods

### 3.1. Chemicals and Reagents

Tris (1-chloro-2-propyl) phosphate (TCPP, 99% purity) was from Dr. Ehrenstorfer GmbH (Augsburg, Germany). The cell counting Kit-8 kit was purchased from GlpBio Technology, Ltd. (Montclair, NJ, USA). The cell cycle testing kit, apoptosis detection kit, SYBR green qPCR master mix, and Total RNA Extraction Reagent were from Yi Fei Xue Biotech Co. (Nanjing, China). The cDNA synthesis kit was from TaKaRa Biotech, Ltd. (Dalian, China). Senescence β-Galactosidase Staining Kit was from Beyotime Biotechnology (Shanghai, China), DAPI was from Sigma Aldrich, and Phosphorylated histone H2A.X (γH2A.X) antibody was from Abcam (Cambridge, UK). Roswell Park Memorial Institute (RPMI) 1640 medium and fetal bovine serum (FBS) were from Procell Life Science & Technology Co., Ltd. (Wuhan, China); Penicillin-streptomycin (PS) and 0.25% trypsin-EDTA were from HyClone (Logan, UT, USA).

### 3.2. Cell Culture and Treatment

The human skin keratinocytes (HaCaTs) were from American Type Culture Collection and cultured with RIPM1640 medium supplemented with 10% FBS and 1% PS in an incubator at 37 °C and 5% CO_2_. HaCaT cells were subcultured twice a week. Before exposure to TCPP, HaCaT cells were seeded in 96-well or 6-well plates overnight. The cells were then treated with 1.56, 6.25, 25, 50, 100, 200, or 400 μg/mL of TCPP for 24 h. TCPP was dissolved in dimethyl sulfoxide (DMSO, Sigma-Aldrich, St. Louis, MO, USA), and the final concentration of DMSO in each treatment was ≤0.1% (*v*/*v*), with 0.1% DMSO solution being set as the control.

### 3.3. Cell Viability Analysis

The effect of TCPP on cell viability was measured using the CCK-8 assay. Briefly, the HaCaT cells were seeded to 96-well plates at the density of 1 × 10^4^ cells/100 μL/well. Following overnight incubation, the cells were treated with different concentrations (1.56, 6.25, 25, 50, 100, 200, or 400 μg/mL) of TCPP for 24 h. Then cells were incubated with 10 μL of CCK-8 solution for 2 h at 37 °C in a 5% CO_2_ incubator. The absorbance at 450 nm was recorded using a microplate reader (Molecular Devices LLC, San Jose, CA, USA). Additionally, the cell morphology was observed by an inverted microscope (TS100, Nikon, Tokyo, Japan). 

### 3.4. Measurement of Intracellular Reactive Oxygen Species (ROS)

The level of intracellular ROS was examined using a ROS assay kit (Yi Fei Xue Biotech Co., Ltd., Nanjing, China) according to the manufacturer’s instructions. Briefly, the HaCaT cells were seeded to 6-well plates and incubated overnight at 37 °C in a 5% CO_2_ incubator at the density of 1 × 10^6^ cells/mL. Based on the median inhibitory concentration (IC_50_) at 275 μg/mL, the cells were treated with TCPP at 1.56–100 μg/for 24 h. Then, the cells were incubated with 10 μM DCFH-DA-containing serum-free medium for 20 min at 37 °C. Cells were washed three times with PBS. Subsequently, the level of intracellular ROS was detected using a flow cytometer (CyFlow^®^Cube 6, Patec, Nuremberg, Germany). Data were collected from 10,000 cells and analyzed by the FlowJo v10.6.2 software (FlowJo LLC, Ashland, OR, USA).

### 3.5. Immunofluorescence Staining

For immunofluorescence staining, the HaCaT cells were seeded on 24-well plates and cultured at the density of 5 × 10^4^ cells/mL overnight; then, the cells were exposed to 1.56–100 μg/mL TCPP for 24 h at 37 °C. Subsequently, HaCaT cells were fixed with 4% paraformaldehyde for 30 min and permeabilized with 0.5% Triton X-100 for 15 min. After being blocked with 1% BSA for 1 h at room temperature, samples were incubated with γH2AX primary antibody (ab81299, abcam, Cambridge, UK, 1:500) overnight at 4 °C. Cells were washed 3 times with ice-cold PBS and incubated with secondary antibodies (ab150077, abcam, Cambridge, UK, 1:200) for 1 h at room temperature. Nuclei were stained blue with 4′,6-diamidino-2-phenylindole (DAPI, Sigma-Aldrich, St. Louis, MO, USA). The fluorescence images were captured with an inverted microscope (Olympus IX73, Tokyo, Japan). The nuclear γH2AX fluorescence intensity was quantified using Image J software (NIH, Bethesda, MD, USA).

### 3.6. Cell Cycle Analysis

For cell cycle analysis, the HaCaT cells were seeded to 6-well plates at the density of 1 × 10^6^ cells/mL. Following overnight incubation, the cells were exposed to 1.56–100 μg/mL TCPP for 24 h, and then they were detected [34]. Briefly, HaCaT cells were collected and fixed in ice-cold 70% ethanol overnight at 4 °C. Afterward, they were incubated with 500 μL staining buffer containing 10 μL of RNaseA and 12.5 μL of propidium iodide (PI) for 30 min at 37 °C in the dark. The cell cycle was analyzed with a CyFlow^®^Cube 6 flow cytometer (Patec, Nuremberg, Germany). Data were collected from 10,000 cells and analyzed by the FlowJo v10.6.2 software (FlowJo LLC, Ashland, OR, USA).

### 3.7. Senescence β-Galactosidase Staining

Senescence β-Galactosidase Staining was performed using the Senescence β-Galactosidase Staining Kit (Beyotime Biotechnology, Shanghai, China) according to the manufacturer’s instructions. Briefly, HaCaT cells were seeded to 6-well plates overnight at 5 × 10^5^ cells/well. After exposing them to TCPP for 24 h, they were washed with PBS and fixed in 1 mL β-galactosidase stain for 15 min at room temperature. Then, they were washed three times with PBS and subsequently incubated overnight with 1 mL staining solution mix (10 μL β-galactosidase staining solution A, 10 μL β-galactosidase staining solution B, 930 μL β-galactosidase staining solution C and 50 μL X-gal solution) at 37 °C. Then, the stained positive cells (blue color) were observed via inverted microscopy (TS-100, Nikon, Tokyo, Japan). The number of SA-β-Gal positive cells was counted using Image Pro-Plus 6.0 software (Olympus, Tokyo, Japan). The percentage of senescent cells was calculated from five individual fields.

### 3.8. Total RNA Extraction and Quantitative Real-Time PCR (qRT-PCR) Assay

After exposure to TCPP for 24 h, the total RNA of HaCaT cells was isolated using TRIzol reagent (Yi Fei Xue Biotech Co., Ltd., Nanjing, China). First-strand cDNA was synthesized with Takara PrimeScript™ RT Master Mix (Dalian, China). The qRT-PCR analysis was performed using an SYBR Green qPCR Master Mix and Roche LightCycler 480II Real-Time PCR system. The amplification conditions were as follows: pre-denaturation at 95 °C for 15 min, followed by 40 cycles of 2-step amplification (95 °C for 15 s and 60 °C for 1 min). The *β-Actin* gene served as an internal control. The relative expression level was calculated by the 2^−ΔΔCT^ method, with primers listed in Table 1.

### 3.9. Enzyme-Linked Immunosorbent Assay (ELISA)

The protein levels of IL-1β and IL-6 in cell supernatants were assayed with human IL-1β and IL-6 ELISA Kits (Yi Fei Xue Biotech Co., Ltd., Nanjing, China) following the manufacturer’s instructions. The absorbance at 450 nm was recorded using a microplate reader (Molecular Devices LLC, San Jose, CA, USA). The results of IL-1β and IL-6 were expressed as concentrations as ng/L.

### 3.10. Statistical Analysis

All experiments were repeated at least three times. Data are presented as the mean ± SEM. All results were analyzed with GraphPad Prism 5.0 statistical software (GraphPad Software Inc., San Diego, CA, USA). Differences in different groups were assessed by one-way ANOVA, with *p* < 0.05 being significant.

## 4. Conclusions

In this study, we found that TCPP caused a concentration-dependent decrease in HaCaT cell viability after exposure to 1.56–400 μg/mL for 24 h, with an IC_50_ of 275 μg/mL, and induced obvious DNA damage and cell senescence. In addition, TCPP induced cell cycle arrest in the G1 phase at 100 μg/mL by upregulation of the mRNA expression of *p53* and *p21*, while the expression of *cyclin D1* was suppressed. Meanwhile, SASP proinflammatory cytokines IL-1β and IL-6 were also enhanced at both mRNA and protein levels. Taken together, our results indicate that TCPP exposure caused cellular senescence may be through the *p53*/*p21* pathway in HaCaT cells, which may provide a new perspective on skin aging.

## Figures and Tables

**Table 1:** The primer sequences used for the RT-qPCR.

Gene	Forward Primer (5′–3′)	Reserve Primer (5′–3′)
*P53*	CAGCACATGACGGAGGTTGT	TCATCCAAATACTCCACACGC
*P21*	TGTCCGTCAGAACCCATGC	AAAGTCGAAGTTCCATCGCTC
*Cyclin D1*	AGCTGTGCATCTACACCGAC	GAAATCGTGCGGGGTCATTG
*CDK4*	AGATGGCACTTACACCCGTG	ACATGTCCACAGGTGTTGCA
*IL-1β*	ACAGATGAAGTGCTCCTTCCA	GTCGGAGATTCGTAGCTGGAT
*IL-6*	CAATCTGGATTCAATGAGGAGAC	CTCTGGCTTGTTCCTCACTACTC
*β-Actin*	GTACCACTGGCATCGTGATGGACT	CCGCTCATTGCCAATGGTGAT

## Data Availability

The data are all presented in this study.
